# Rescue thrombolysis for medium vessel occlusion (RESCUE-TNK): Rationale and design of a phase 2 randomized trial

**DOI:** 10.3389/fneur.2023.1154736

**Published:** 2023-03-27

**Authors:** Hai-Zhou Hu, Jing Qiu, Wei Li, Thanh N. Nguyen, Feng Wang, Di Li, Huai-Zhang Shi, Shou-Chun Wang, Ming Wei, Hui-Sheng Chen

**Affiliations:** ^1^Department of Neurology, General Hospital of Northern Theatre Command, Shenyang, China; ^2^Neurology, Radiology, Boston Medical Center, Boston, MA, United States; ^3^Department of Interventional Therapy, The First Affiliated Hospital of Dalian Medical University, Dalian, China; ^4^Neurological Intervention Department, Dalian Municipal Central Hospital, Dalian, China; ^5^Department of Neurosurgery, First Affiliated Hospital of Harbin Medical University, Harbin, China; ^6^Department of Neurology, First Affiliated Hospital of Jilin University, Changchun, China; ^7^Department of Neurosurgery, Tianjin Huanhu Hospital, Tianjin, China

**Keywords:** rescue thrombolysis, medium vessel occlusion, tenecteplase, rational, design

## Abstract

**Background:**

The best reperfusion strategy for medium-sized vessel occlusion (MeVO) is not well established. Given the proven treatment effect of intra-arterial thrombolysis in patients with large vessel occlusion (LVO), we hypothesized that intra-arterial tenecteplase (TNK) could increase the recanalization rate of MeVO and thus improve clinical outcome.

**Aims:**

To explore the safety and efficacy of intra-arterial TNK in patients with MeVO.

**Sample size estimates:**

A maximum of 80 patients are required to test the superiority hypothesis, using power = 80% and *α* = 0.025 to conduct the one-sided test.

**Design:**

Rescue treatment for mEdium veSsel oCclUsion by intra-artErial TNK (RESCUE-TNK) is a pilot, randomized, open-label, blinded end point, and multicenter trial. Eligible patients including primary MeVO as detected by the first DSA examination or secondary MeVO after endovascular treatment (EVT) for LVO will be assigned into the experimental group and control group as a ratio of 1:1. The experimental group will be treated with intra-arterial TNK (0.2–0.3 mg/min, for 20–30 min) *via* a microcatheter placed proximal to the site of occlusion, and the control group will be treated with routine therapy. Both groups of patients will be given standard stroke care based on the guidelines.

**Outcome:**

The primary efficacy end point is successful recanalization of MeVO, defined as the expanded treatment in cerebral ischemia (eTICI) score 2b67-3 after the procedure, while the primary safety end point is symptomatic intracranial hemorrhage (sICH), defined as National Institutes of Health Stroke Scale score increase ≥4 caused by intracranial hemorrhage within 24 (−6/+24) hours after randomization.

**Conclusion:**

The results of RESCUE-TNK will provide evidence for the efficacy and safety of intra-arterial TNK in the recanalization of patients with MeVO.

## Introduction and rationale

Reperfusion of an occluded artery is the primary treatment goal in acute ischemic stroke (AIS) to achieve good clinical outcomes. Endovascular treatment (EVT) is recommended as the standard of care in patients presenting with acute large vessel occlusion (LVO). (1) Medium-sized vessel occlusion (MeVO), usually defined as occlusions of the M2-3, A1-3, P1-3, anterior inferior cerebellar artery (AICA), posterior inferior cerebellar artery (PICA) or superior cerebellar artery (SCA) segment, accounts for 25–40% of AIS, (2) mainly involving primary isolated MeVO or secondary embolism caused by EVT or intravenous thrombolysis (IVT). Due to a more distal site of arterial occlusion and a relatively smaller infarct size, the prognosis of MeVO stroke is generally better than LVO. However, the prognosis remains poor in many patients, (3) particularly in those with primary M2-3, A1, P1 MeVO or secondary MeVO including emboli to new territories and emboli to distal territories in the setting of EVT. (4)

In patients with disabling MeVO, treatment decision-making can be a challenge for physicians. Some studies have shown that EVT for MeVO is safe and may confer benefit, (5–7) while other studies have reported a high risk of intracranial hemorrhage (ICH). (8, 9) In addition, EVT for MeVO is restricted by many factors, including relatively greater anatomical tortuosity to reach the target site of occlusion, the need for lower-profile devices for smaller arteries, and operator skill. Among neurointerventionalists, the inclination for treating MeVO with a mechanical device is significantly greater in patients who are ineligible for IVT compared to those who are IVT eligible. (10) Thus, the treatment for MeVO in clinical practice will vary according to the patient’s eligibility for IVT, different centers, or operators. In PROACT-II (11), which demonstrated the efficacy and safety of local thrombolysis with prourokinase for LVO, intra-arterial prourokinase increased partial or complete reperfusion rates among 44 patients with isolated M2 occlusions (12). This was also confirmed in another study demonstrating greater reperfusion rates with rescue intra-arterial urokinase after failed or incomplete reperfusion with mechanical thrombectomy. (13)

As the latest generation of thrombolytic drugs, tenecteplase (TNK) has been found to be associated with higher reperfusion rates and better functional outcome than alteplase in bridging EVT patients with large vessel occlusion both in anterior and posterior circulation, (14) although this was not confirmed in the ACT trial (15). In our recent unpublished study, intra-arterial TNK (12 mg) was used during mechanical thrombectomy for large vessel occlusion patients and found the treatment did not increase the risk of cerebral hemorrhage.

In this context, we hypothesized that intra-arterial TNK could increase the recanalization rate and thus improve clinical outcomes in MeVO patients who are considered ineligible for mechanical clot retrieval. Therefore, the purpose of this study is to explore the safety and efficacy of this treatment strategy.

## Methods

### Design

**Figure 1:**
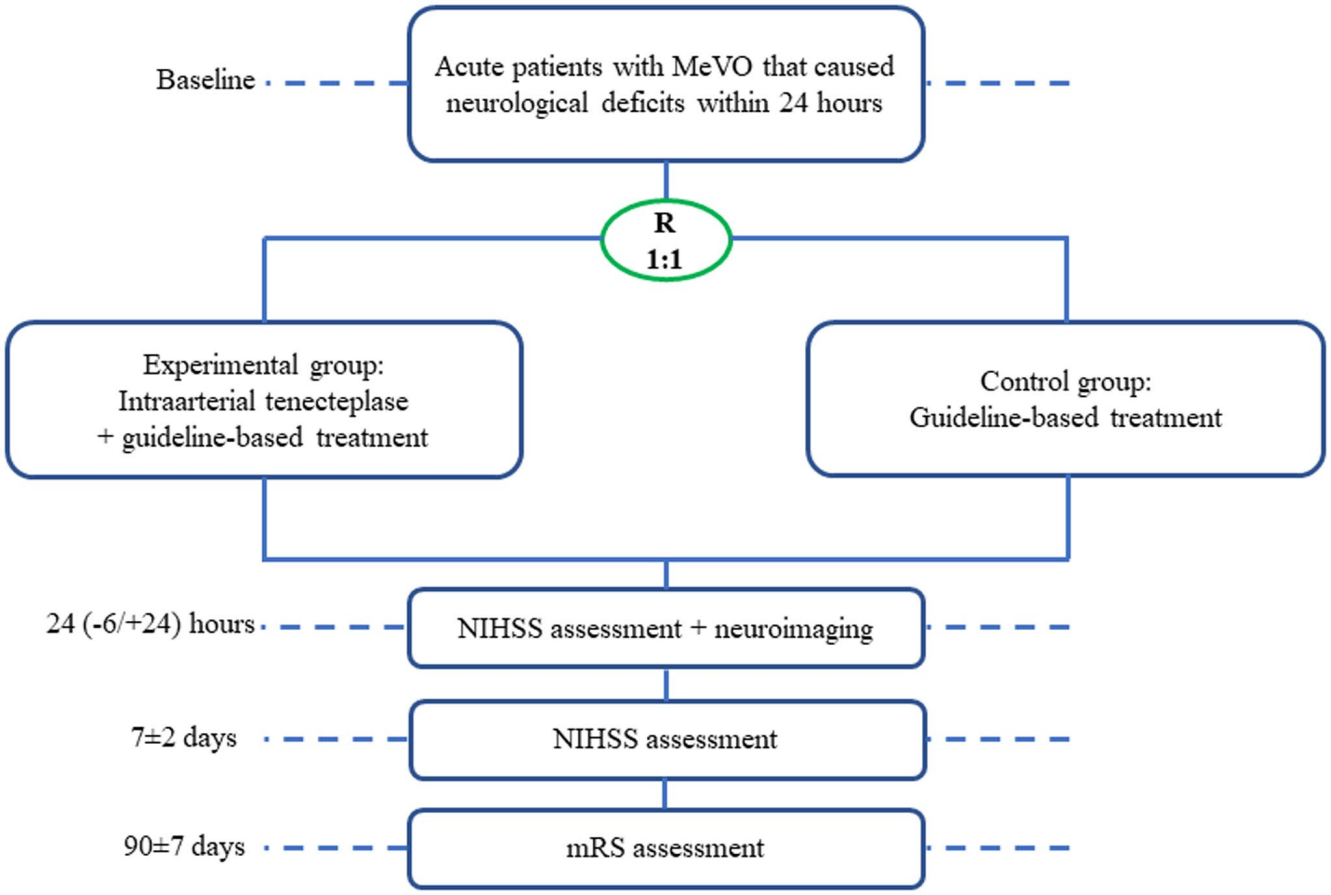
Trial flowchart. R, randomization; NIHSS, national institutes of health stroke scale; mRS, modified ranking scale.

Rescue treatment for mEdium veSsel oCclUsion by intra-artErial TNK (RESCUE-TNK) is a pilot, randomized, open-label, blinded end point, and multicenter study in China aiming to evaluate the safety and efficacy of intra-arterial TNK in patients presenting with MeVO. The trial flowchart is shown in Figure 1. For primary MeVO, CTA or MRA will be usually used to screen the eligibility, which will be confirmed by DSA; for secondary MeVO, DSA will be usually screened and confirmed for the eligibility.

### Study population

In the RESCUE-TNK trial, eligible participants are AIS patients with primary MeVO detected by the first DSA, or secondary MeVO after mechanical thrombectomy. Patients will be enrolled at approximately 20 sites in China between March 2023 and March 2025. The detailed inclusion/exclusion criteria are listed in Table 1. Specifically, intravenous thrombolysis should be administrated within 270 min according to current guidelines.

**Table 1:** Inclusion/exclusion criteria.

Inclusion criteria:
Age ≥ 18 years;
Medium vessel occlusion (MeVO), referring to M2-3 of MCA; A1-3 of ACA; P1-3 of PCA; PICA, AICA or SCA (including primary, distal embolism in the same region after thrombectomy or concurrent embolism in other regions).
Primary MeVO as detected by the first DSA examination or secondary MeVO after mechanical thrombectomy for large vessel occlusion;
MeVO causes neurological deficits in motor strength, language, vision etc.;
Endovascular mechanical thrombectomy cannot be performed as assessed by the investigator;
Absence of parenchymal hematoma on CT images performed in the angio suite.
Within 24 h from symptom onset;
Signed informed consent by patient or patient’s legally authorized representative.
Exclusion criteria:
Patients with completed infarction in the territory of the MeVO on non-contrast CT;
Patients with intracranial hemorrhage;
Coagulation disorders, tendency for systemic hemorrhagic, thrombocytopenia (<100,000/mm^3^);
Severe hepatic or renal dysfunction, increase in ALT or AST (more than 2 times of upper limit of normal value), increase in serum creatinine (more than 1.5 times of upper limit of normal value) or requiring dialysis;
After mechanical thrombectomy, severe and sustained (> 5 min) uncontrolled hypertension (systolic blood pressure over 180 mmHg or diastolic blood pressure over 105 mmHg);
Patients with contraindication or allergy to any ingredient of study medication;
Pregnancy, plan to get pregnant or active lactation;
The estimated life expectancy is less than 6 months due to other serious diseases;
Other conditions unsuitable for this clinical study as assessed by researcher.

### Standard protocol approvals, registrations, and patient consents

The RESCUE-TNK trial is listed on www.clinicaltrial.gov (NCT05657470). The protocol and data collection of the trial will/have been approved by the ethics committee of the General Hospital of Northern Theater Command and all participating sites. All patients or their representatives will provide written informed consent before inclusion into the trial.

### Randomization and intervention

Eligible patients will be assigned into an experimental group and a control group as a 1:1 ratio by using central and computerized random sequence generation (dynamic randomization-minimization) as stratified by center. The experimental group will be treated with intra-arterial (IA) TNK *via* a microcatheter proximal to the site of the occlusion for 20 to 30 min. TNK will be temporarily stopped at 20 min from the onset of infusion, at which time recanalization status will be adjudicated by DSA. If successful recanalization is not achieved, TNK will be administered for the remaining 10 min. Different doses of intra-arterial TNK will be given based on whether intravenous thrombolysis or rescue balloon angioplasty and/or stenting is performed: (1) 0.3 mg/min IA TNK if neither treatment was given; (2) 0.25 mg/min IA TNK if either of them was given; (3) 0.2 mg/min IA TNK if both treatments were given. The control group will be treated with routine therapy. Both groups of patients will be given standard care based on the guidelines. (16, 17)

During the first 24 h, we recommend that SBP will be tightly controlled to less than 180/100 mmHg and less than 140/90 mmHg if eTICI ≥2b67 is achieved; blood glucose level will be controlled to less than 160 mg/dl.

### Outcomes

The primary efficacy end point is proportion of patients with successful MeVO recanalization, defined as the expanded treatment in cerebral ischemia (eTICI) score 2b67-3 in the territory of the target occluded MeVO artery. (18)

The secondary efficacy end points are as follows: (1) proportion of modified Rankin Scale (mRS) 0–1 at 90 days; (2) proportion of mRS (0–2) at 90 days; (3) distribution of mRS at 90 days; (4) incidence of early neurological improvement (ENI), defined as a decrease of more than four points or reaching zero points in NIHSS score, compared with baseline at 24 (−6/+24) hours; (5) change in NIHSS compared with baseline at 24 (−6/+24) hours; (6) rate of visual recovery in patients with posterior cerebral artery occlusion at 7 days or at hospital discharge, whichever is earlier (categorized as no recovery, partial recovery and complete recovery).

The primary safety end point is the proportion of symptomatic intracranial hemorrhage (sICH) within 24 (−6/+24) hours, which is defined as an increase in the NIHSS score of ≥4 points as a result of the intracranial hemorrhage (19).

The secondary safety end points include proportion of parenchymal hematoma type 1 and 2 (PH1 and PH2) at 24 (−6/+24) hours, all serious adverse events at 24 (−6/+24) hours, all-cause death within 7 ± 2 days or during hospitalization and proportion of recurrent stroke, cardiovascular events, other vascular events and death within 90 days.

### Follow-up procedure

Study visits will be performed at 24 (−6/+24) hours, 7 ± 2 days, and 90 ± 7 days after randomization (Figure 1). At baseline, demographic characteristics, pre-stroke mRS, NIHSS, routine laboratory tests, and neuroimaging including CT perfusion data if performed will be recorded. At 24 h, a repeat non-contrast CT + CTA/MRI + MRA will be performed for both groups. NIHSS scoring will be repeated at 24 (−6/+24) hours, 7 ± 2 days, and mRS will be assessed at 90 ± 7 days. All clinical assessments including NIHSS and mRS will be evaluated by certified assessors according to a standardized procedure manual at each site. An anterior-posterior (AP) and the lateral view will be obtained on all angiographic runs for the site of MeVO occlusion at baseline and the end of the procedure. The primary endpoint (eTICI scores) will be initially assessed by the local investigator and later adjudicated by a central neuroimaging lab blinded to treatment allocation. To prevent bias, the 90-day clinical assessments including mRS will be evaluated by one qualified personnel blinded to treatment allocation. To ensure the validity, consistency, and reproducibility of the evaluation, we will have a training course for all investigators at each center. MRS at 90 days will be evaluated in person or by telephone interview if an in-person evaluation is not possible. Adverse events within 90 days of randomization will be recorded in detail by investigators. The cycle of this research is expected to be approximately 24 months.

### Data management and quality control

All data will be obtained by MedSci^1^ which includes the patient case report form. The data will be downloaded from MedSci with a dedicated person for statistical analysis. The data safety and monitoring committee (DSMC) will perform data review to monitor safety outcomes, such as hemorrhagic events and other adverse events (AEs), etc. A central core lab for imaging was set to judge intracranial bleeding events and assess neuroimaging data. An interim analysis will be conducted after 50% of patients have had their 90-day follow-up by an independent statistician of the DSMC, who is not involved in managing the trial. Based on this analysis, the DSMC can recommend to the Steering Committee to adjust the sample size or early termination of the study. As for the interim analysis, no formal analysis of the primary endpoint will be performed, to avoid the occurrence of a type-1 error in the final analysis.

### Adverse event monitoring

An AE is any adverse medical event that occurs during the study. All information about AEs should be recorded on the AE page of the case report, and whether the unexpected AE is associated with intra-arterial TNK treatment will be further adjudicated by the DMSB.

### Sample size determination

Subgroup analysis of PROACTII found that the reperfusion rate of intra-arterial thrombolysis for M2 occlusion was 53.6%, while that of the control group was 16.7%, (12) with a difference of 36.9%. Accordingly, we estimate that the proportion of recanalization of MeVO in the control group in this study will be 20%, while the proportion in the experimental group is conservatively estimated to be 50%, with a 30% increase. Using a power of 80% and *α* of 0.025 to carry out the one-side test, the calculated sample size is 72. In consideration of the 10% lost to follow-up, the total sample size is 80. Therefore, this study will include 80 patients, with 40 patients in each group.

### Statistical analysis

An intention-to-treat (ITT) analysis will be used to analyze the therapeutic effects of the two groups and all data will be analyzed with SPSS 20.0 Software. The mean ± standard deviation (SD) will be used if the data are normally distributed; the median and interquartile range (IQR) will be used if the data are not normally distributed. Differences in the proportion of successful recanalization of MeVO (eTICI score 2b67-3 for the target vessel), mRS (0–1) at 90 days, mRS (0–2) at 90 days, and ENI will be compared using binary logistic regression. The distribution of the mRS at 90 days between the two groups will be compared using ordinal logistic regression. Time-to-events of stroke recurrence and other vascular events will be compared using Cox regression. The change in NIHSS score within 48 h between the two groups will be compared using a general linear model. All statistical tests are 2-sided, using *α* = 0.05 and *p* < 0.05 as considered statistically significant.

The subgroup for primary endpoint will be performed by age, sex, different doses of TNK, type of MeVO, NIHSS score at presentation, time from onset to randomization, whether intravenous thrombolysis, stroke territory, and stroke etiology.

### Study organization and funding

The protocol was designed by Hui-Sheng Chen and discussed by the trial steering committee. The trial steering committee is comprised of external scientific advisors and will organize teleconference or physical meetings to provide recommendations about the trial. Neuroimaging associated with clinical events will be collected centrally and interpreted by a central neuroimaging lab including two independent neuroradiologists. The trial is initiated by the Cerebrovascular Disease Collaboration & Innovation Alliance (CDCIA) of Liaoning and supported by grants from the Science and Technology Project Plan of Liao Ning Province.

### Current status

At the time of the first submission of this article, the trial has not begun to recruit patients. Recruitment will be continued until the complete sample size is achieved, which is expected to be until January 2024.

## Discussion

As an effective treatment for LVO stroke, EVT is highly recommended by current guidelines. Some studies suggested the feasibility, safety, and possible benefit of EVT for MeVO, but the treatment may vary according to the patient’s anatomy, location, type of occlusion, available devices, and neurointerventionalist’ skills. Rescue intra-arterial thrombolysis has been found to be safe and effective for LVO, but its efficacy for opening a MeVO is unknown. Collectively, the ideal recanalization strategy for MeVO is unclear. (4, 20)

RESCUE-TNK is a prospective multicenter randomized trial investigating the efficacy and safety of intra-arterial TNK in patients with MeVO. In contrast to prior studies, this trial has several distinct characteristics. First, previous studies on MeVO reperfusion strategy mainly focused on mechanical thrombectomy, (4–9, 20, 21) with little regard to the effect of intra-arterial thrombolysis, which will be investigated in this trial. Second, most studies were single-center and retrospective, (22–24) while the prospective multicenter design is being used in this trial. Third, the M2 site of occlusion has been widely studied in previous MeVO studies, (7–9, 12) while this trial will enroll all MeVO patients, which will allow the study to be more inclusive of patients with medium or distal vessel occlusion in different locations. (25) The lack of requirement of perfusion imaging to select patients in this trial may permit the results to be more generalizable to centers that do not routinely use advanced imaging in their selection of patients for EVT. (25, 26) Fourth, TNK for intra-arterial thrombolysis will be used in this trial, given that TNK has greater fibrin specificity, higher reperfusion rates, and possibly better safety profile than alteplase. (27) Fifth, we did not set the lower limit of the baseline NIHSS score, given the disability of some MeVO patients with low NIHSS score (such as isolated aphasia and hemianopia), and early successful recanalization should be beneficial for this population who was judicated by local investigator. Finally, the unique intra-arterial thrombolysis strategy with doses adjusted to the patient’s clinical presentation will be used in this trial to balance the benefit and risk.

## Conclusion

The results of RESCUE-TNK will provide high-quality evidence for the efficacy and safety of intra-arterial TNK in patients with primary or secondary MeVO.

## Ethics statement

The studies involving human participants were reviewed and approved by the ethics committee of the General Hospital of Northern Theater Command. The patients/participants provided their written informed consent to participate in this study.

## Author contributions

H-ZH and JQ wrote the draft. WL, TN, FW, DL, H-ZS, S-CW, and MW critically revised the manuscript. H-SC designed the manuscript and critically revised the manuscript. All authors contributed to the article and approved the submitted version.

## Funding

This work was supported by grants from the Science and Technology Project Plan of Liao Ning Province (2022JH2/101500020).

## Conflict of interest

The authors declare that the research was conducted in the absence of any commercial or financial relationships that could be construed as a potential conflict of interest.

## Publisher’s note

All claims expressed in this article are solely those of the authors and do not necessarily represent those of their affiliated organizations, or those of the publisher, the editors and the reviewers. Any product that may be evaluated in this article, or claim that may be made by its manufacturer, is not guaranteed or endorsed by the publisher.

## Footnotes

^1^ http://BRETIS-TNK2.medsci.cn
