# Supplementary material for: Rescue thrombolysis for medium vessel occlusion (RESCUE-TNK): Rationale and design of a phase 2 randomized trial
Source: Front Neurol. 2023 Mar 27;14:1154736. doi: 10.3389/fneur.2023.1154736 (PMC10084791; doi:10.3389/fneur.2023.1154736)
Supplement: Supplementary file 1 [file Data_Sheet_1.docx]

**Supplemental files for the telephone interview case report form (CRF) and standard operating procedure (SOP) of modified Rankin Score**

**Appendix 1：****CRF of modified Rankin Score**

| Grade | Description | Actual score |
| --- | --- | --- |
| 0 | No symptoms |  |
| 1 | Symptoms without any incapacity (able to perform all usual activities) |  |
| 2 | Mild incapacity (unable to perform all usual activities but able to look after his/her affairs alone) |  |
| 3 | Moderate incapacity (requires assistance but walks alone) |  |
| 4 | Severe incapacity (requires assistance for walking and physical body needs) |  |
| 5 | Severe incapacity (bedbound, incontinent, permanent surveillance required) |  |
| 6 | Death |  |

**Appendix 2：The SOP of the telephone interview for modified Rankin Scale (mRS)**

- **mRS assessors:** 1-2 investigators will be authorised as blinded mRS assessors in each site.
- **The training of modified Rankin Scale (mRS) assessment:** the blinded mRS assessors were trained uniformly on how to evaluate the mRS based on face to face or a structured telephone interview algorithm. After the training, 20 examples based on the neurological function description of patients were used to assess this score and assessors were certified when intraclass correlation coefficient ≥ 0.95.
- **Structure interview for telephone assessment:** a structured telephone interview and interview algorithm was used as reported in a previous study, in which there was consistency and reproducibility of the mRS assessment between telephone and in-person interviews. (Isaksson et al., Eur Neurol. 2020. DOI: 10.1159/000510721).
